# Long covid and general practice: a survey of patients in the Republic of Ireland

**DOI:** 10.1007/s11845-026-04481-1

**Published:** 2026-06-13

**Authors:** John Broughan, Yao Xie, Crea Carberry, Sarah O’Connell, Jacinta Fay, Pamela Morrison, Nandakumar Ravichandran, Stefano Savinelli, Geoff McCombe, Mary Higgins, John S. Lambert, Walter Cullen

**Affiliations:** 1 Clinical Research Centre, School of Medicine, University College Dublin, Dublin, Ireland; 2 School of Medicine, University College Dublin, Dublin, Ireland; 3 Long Covid Advocacy Ireland, Dublin, Ireland; 4 St Vincent’s University Hospital, Dublin, Ireland; 5 National Maternity Hospital, Dublin, Ireland; 6 Mater Misericordiae University Hospital Dublin, Dublin, Ireland; 7 The Rotunda Hospital, Dublin, Ireland

**Keywords:** COVID-19, Cross-Sectional Studies, General Practice, Long Covid, Primary Health Care, Surveys and Questionnaires

## Abstract

**Background:**

Long Covid is a multi-factorial condition impacting millions globally.

**Aims:**

(1) To describe the demographic and health profiles of adults who have or have had Long Covid, and (2) examine their experiences of Long Covid healthcare, particularly in general practice. A cross-sectional online survey was conducted in the Republic of Ireland in April 2025.

**Methods:**

Survey items investigated demographics, medical history, and experiences of Long Covid care. Data was analysed using descriptive statistics and qualitative analysis of open responses.

**Results:**

Of 222 (87.05%) eligible respondents, most were 35–64 years (n = 184, 82.14%), female (n = 177, 79.7%), and born in the Republic of Ireland (n = 193, 86.9%). Respondents reported wide-ranging multimorbidity and Long Covid symptoms. Many strongly agreed that their symptoms were severe (n = 196, 88.7%) and detrimental to quality of life (n = 200, 90.1%), physical (n = 202, 91%) and mental (n = 98, 45%) health. Long Covid commonly lasted more than three years (n = 169, 76.1%), indicating substantial long-term impact on daily functioning. Almost all consulted general practitioners for Long Covid care (n = 213, 95.9%). Nearly half (n = 95, 44.6%) were dissatisfied with current GP care, and many felt GPs lacked adequate knowledge of Long Covid (n = 127. 59.6%). Respondents supported proposed GP-based investigative and referral interventions, as well as affirming and advocacy-focused doctor–patient relationships.

**Conclusions:**

Continued clinical and research efforts are needed to address future Long Covid care needs. With structured, informed, holistic, and multidisciplinary care, general practice can make a positive contribution to the lives of those affected.

**Supplementary Information:**

The online version contains supplementary material available at https://doi.org/10.1007/s11845-026-04481-1.

## Introduction

Long Covid is a post-viral condition that can result following a SARS-CoV-2 infection, the virus causing COVID-19. There are many symptoms a person living with Long Covid can experience; the World Health Organization (WHO) has documented more than 200 [1]. Symptoms are also highly variable, and their nature can differ considerably from person to person, whether that be in terms of the kinds of symptoms people have, the amount of time symptoms last for, and their severity. Also, like other chronic conditions, Long Covid can have debilitating indirect consequences on health, functioning, and wellbeing for many people [2–4]. Globally, it is still unclear what the prevalence of Long Covid is today or has been in the years following the COVID-19 pandemic. For instance, a 2023 epidemiological review of international evidence by Ireland’s Health Information and Quality Authority (HIQA) reported Long Covid prevalence estimates ranging between 1.8 and 53.1% [5]. This large discrepancy is due to numerous factors including variations in Long Covid definitions used, study designs, and clinical recording practices [6]. The same report estimated Long Covid prevalence in Ireland at 16%. Long Covid has shown to be most common among people in middle age, females, those with existing multimorbidity, people that experienced severe and/or complex COVID-19 symptoms, health and social care workers, people from disadvantaged socioeconomic backgrounds, and racial/ethnic minorities [6–8].

In the early stages of the COVID-19 pandemic, the Long Covid phenomenon was met with great public concern; mostly due to the then widespread presence of risk factors such as the high incidence and severity of COVID-19 strains and the absence of a COVID-19 vaccine [9]. Fortunately, considerable progress has since been made to address these issues, with research indicating that Long Covid’s epidemiological risk has largely declined [10, 11]. Despite this, Long Covid has not disappeared, and many people remain affected [3]. Estimates vary widely due to differences in definitions and diagnostic criteria. For example, a 2023 Swedish cohort study using ICD-10 codes reported a prevalence as low as 2%, while a 2025 US study estimated incidence between 10–26% in adults with prior COVID-19 [12, 13]. Patients commonly present in primary care and are often referred to specialist or multidisciplinary services, reflecting the persistent, multi-system nature of Long Covid [1].

Affected individuals still need care, and reduced access to appropriate health and social services for this cohort, such as integral community health services like general practice, would be detrimental for their long-term health and wellbeing. Given the varied nature of Long Covid symptoms and its chronic nature, holistic, multidisciplinary, well integrated, and continuous health supports are recommended [14–16]. As experts in providing supports of this nature, primary care physicians, particularly general practitioners (GPs), are well suited to caring for patients living with Long Covid, as is the case for many other long-term and chronic health conditions [2, 4, 17–21]. Previous research on GP Long Covid care has highlighted the importance of sufficient Long Covid knowledge among GPs, standardised care processes, (particularly around diagnosis, assessment, monitoring, and referrals), integrated multidisciplinary care approaches, and the importance of establishing a supportive and collaborative doctor-patient relationship [2, 17, 22–24].

Understanding the patient’s perspective is essential to ensure future health interventions are person-centred. Going forward, acquiring and acting upon stakeholders’ perspectives (e.g., patients, primary and secondary care workers, carers), namely their accounts of prior care experiences and recommendations for future practice, is key to making sure that general practice maximises its already advantageous position in the realm of Long Covid care [25]. Therefore, this study was conducted with people in the Republic of Ireland that have, or have had, Long Covid to answer the questions:

1. What are the demographic and medical characteristics of general practice patients living with Long Covid?
2. Is general practice well suited to delivering Long Covid care?
3. How can GPs improve Long Covid care and care outcomes going forward?

## Methods

### Study design

A cross-sectional online survey design informed by the STROBE guidelines [24] was used to provide a snapshot of people living with Long Covid in the Republic of Ireland in 2025, and to investigate their perspectives regarding Long Covid and its management in general practice. As per the Medical Research Council’s ‘Framework for the Development of Complex Interventions’, a model which continually informs our work towards developing GP based Long Covid interventions, study aims and methodology were informed by the findings of our previous research on the topic [2, 17] and consultation with healthcare providers and patient stakeholders.

### Sampling

People were eligible to participate in the study if they:

- Were aged ≥ 18 years
- Were resident in the Republic of Ireland
- Self-reported having previously received a positive COVID-19 Polymerase Chain Reaction (PCR) or Antigen test or having previously been clinically diagnosed by a qualified health professional with COVID-19.
- Believed that (1) they have, or have in the past, experienced long-term health problems resulting from COVID-19 infection or (2) been diagnosed with Long Covid by a qualified health professional.

### Recruitment and procedure

Electronic invitations to participate in the survey were distributed through the study investigators’ social media channels (X/Twitter) and professional networks, as well as through the channels, networks and the mailing list of a Long Covid advocacy group in Ireland. Potential participants could click on a Google Forms survey link and read the study participant information leaflet (PIL) and consent form. To complete the survey, potential participants were first required to provide informed consent by ticking boxes indicating that they read the PIL, understand what the study involves (i.e., its potential risks, who to contact if they have queries, that they are free to withdraw from the study any time they wish), and are happy to take part. Participants commenced taking the survey once consent was obtained.

### Data collection

Data collection spanned from the 1st to 20th April 2025. The survey investigated participant demographics (e.g., age, gender); history of COVID-19, Long Covid, and other conditions if relevant; access to healthcare, and personal experiences and views regarding the management of Long Covid in healthcare settings, particularly in general practice. All the survey questions are listed in the accompanying supplementary material.

### Data analysis

Descriptive statistics (frequencies & percentages) were employed to analyse survey responses using SPSS version 29 [26]. Qualitative analysis using NVivov20 was applied to summarise responses to open-ended questions.

## Results

Characteristics of the study’s participants are available in Table 1 below.

**Table 1:** Sample characteristics

		Count (n)	Percentage (%)
Total Responses	All Survey Submissions	255	100%
Inclusion Status	Eligible for Analysis	222	87.05%
	Ineligible (Total)	33	12.94%
Reasons for Ineligibility	Not a resident of ROI	17	6.70%
	No response to eligibility	9	3.53%
	Under 18 years old	5	2.00%
	Never had COVID-19	2	0.80%
Age	Primary Range (35–64)	184	82.14%
	Peak Subgroup (45–54)	85	38.30%
Gender	Female	177	79.70%
	Male	41	18.50%
	Non-binary	3	1.40%
	Prefer not to say	1	0.50%
Country of Birth	Republic of Ireland	193	86.90%
	Other EU	15	6.80%
	Non-EU	14	6.30%

### History of COVID-19, long covid and other conditions

**Fig. 1:**
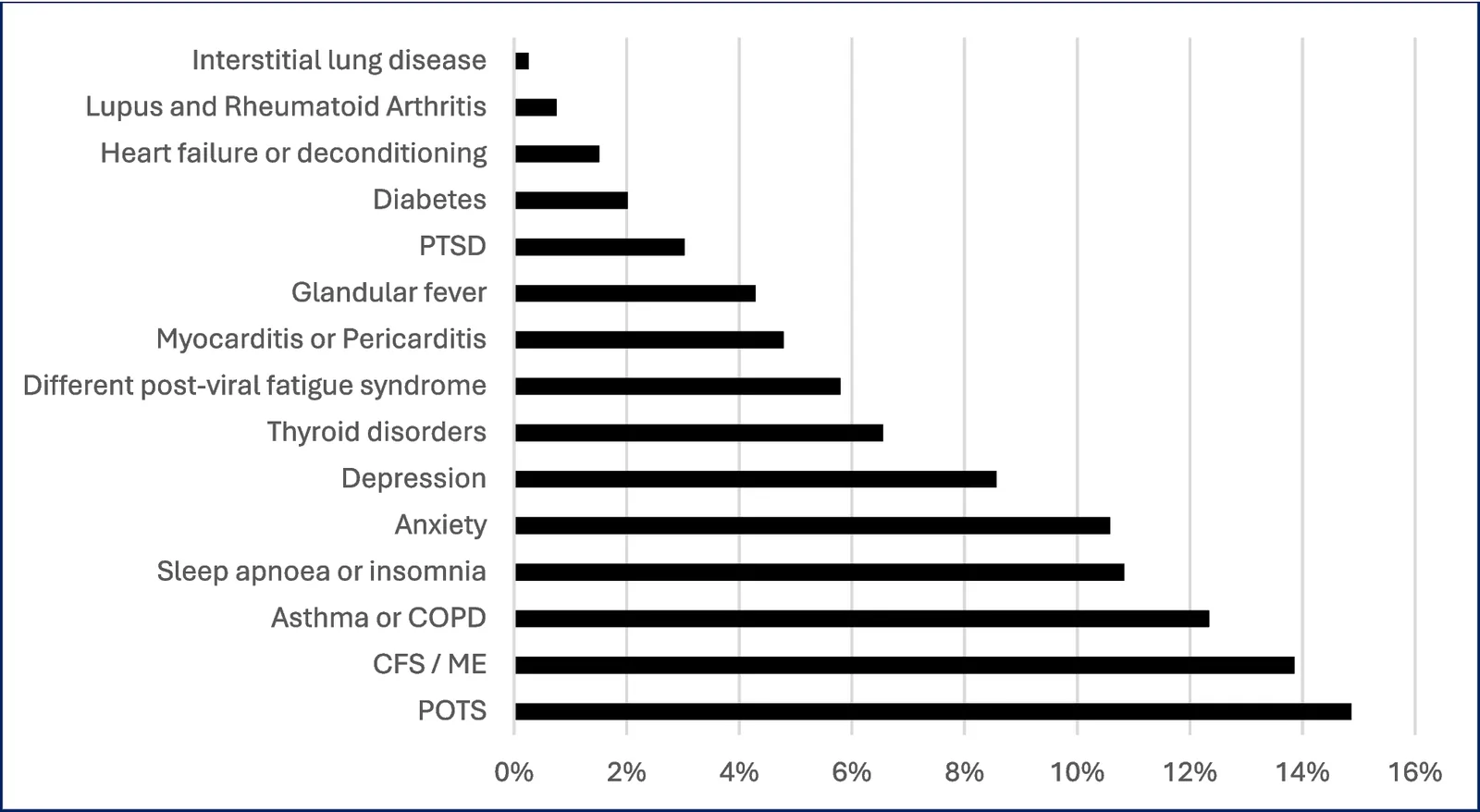
Percentage distribution of past and present ‘other diagnoses’ among the sample (n = 222). PTSD: Post Traumatic Stress Disorder. CFS: Chronic Fatigue Syndrome. ME: Myalgic Encephalomyelitis. POTS: Postural Orthostatic Tachycardia Syndrome

Most respondents said they have had COVID-19 one to three times (n = 195, 87.8%), with similar numbers evident among those who had it once (n = 60, 27%), twice (n = 79, 35.6%), and three times (n = 56, 25.2%). Two hundred and eight (94.5%) had COVID-19 diagnoses confirmed by a PCR/Antigen test or a qualified health professional. When asked *“Who raised the possibility that you might have Long Covid?”*, most respondents said a health professional (n = 116, 52.3%) or that they raised this possibility themselves (n = 84, 37.8%). One hundred and ninety-five (87.8%) respondents’ Long Covid diagnoses were confirmed by a qualified health professional. Most respondents reported that their first COVID-19 infection triggered Long Covid (n = 164, 73.9%). Respondents also reported having been diagnosed with a wide range of other health conditions (Fig. 1). One hundred and fifteen (52%) respondents reported that health professionals suggested that their physical symptoms might be caused by psychological issues such as stress and anxiety.

### Symptoms of COVID-19/long covid and their impact

**Fig. 2:**
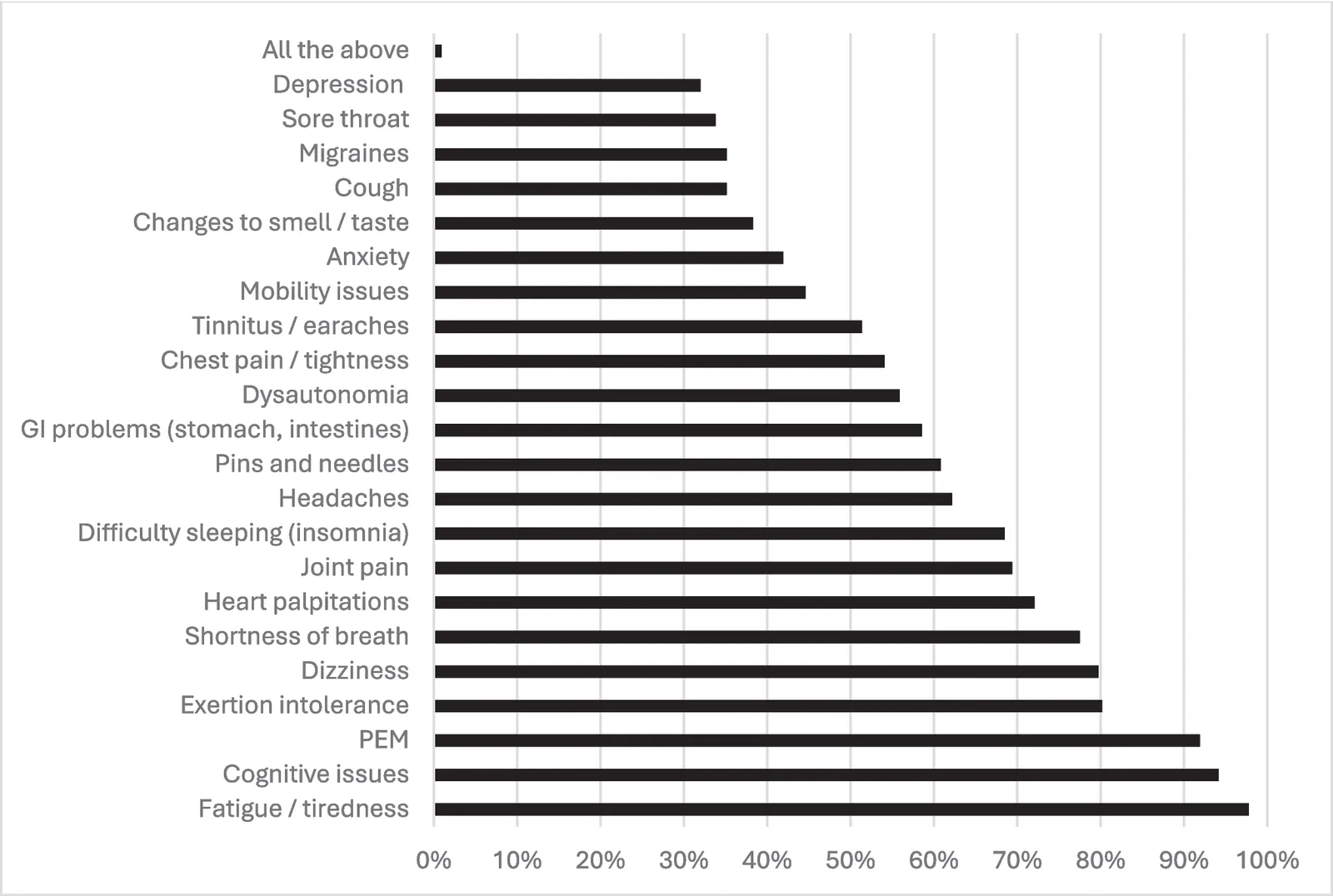
Symptoms experienced due to Long Covid (n = 222). GI problems: Gastrointestinal problems. PEM: Post Exertional Malaise (extreme fatigue after activity)

Most respondents agreed (n = 57, 25.8%) or strongly agreed (n = 140, 63.3%) that the first two to three weeks of having COVID-19 made them feel unwell. Only 12 (5.4%) respondents disagreed or were neutral in this regard. Most (n = 196, 88.7%) strongly agreed that having Long Covid made them feel unwell. When asked to indicate the approximate length of time that they have experienced Long Covid issues, most (n = 102, 45.9%) respondents said more than four years, with more than three years (n = 67, 30.2%) and more than two years (n = 36, 16.2%) the next most common responses. Most respondents strongly agreed that Long Covid negatively impacted their physical health (n = 202, 91%) and quality of life (n = 200, 90.1%), and most strongly agreed (n = 98, 45%) or agreed (n = 73, 33.5%) that it negatively impacted their mental health. The percentage distribution of symptoms respondents experienced due to Long Covid is in Fig. 2. Open responses described Long Covid’s physical health impacts as severe and disabling (n = 198), its impacts on quality of life as significantly disruptive to the physical, social, emotional and economic parts of life (n = 192), and its mental health impacts as profound, distressful, and laden with persistent low mood, anxiety, isolation, and loss of identity (n = 173).

### Long Covid related general practice (and other healthcare) experiences

**Fig. 3:**
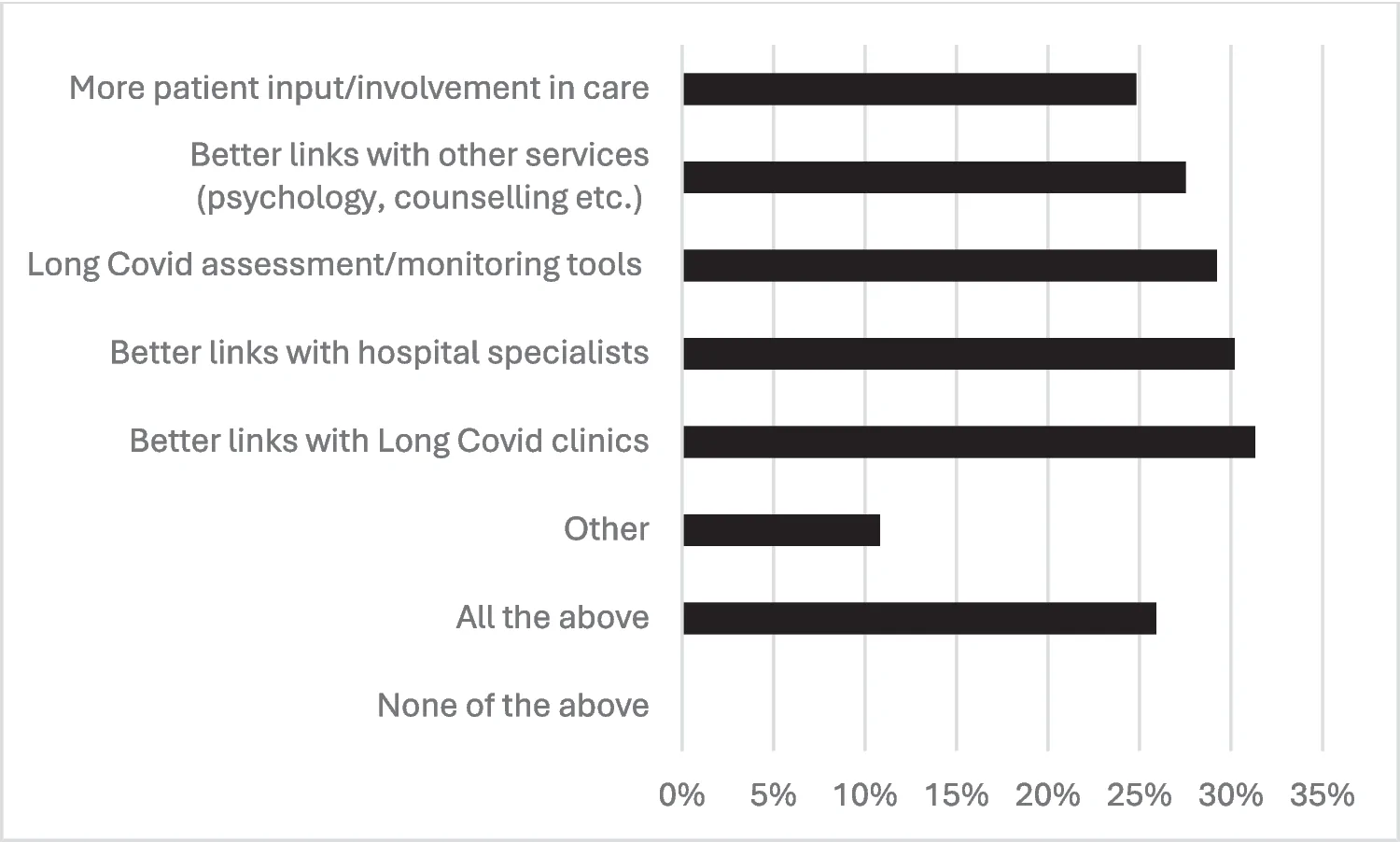
“Which of the following initiatives would you like to see introduced by general practitioners for care of patients with Long Covid problems?” (n = 219)

Two hundred and thirteen (95.9%) respondents said they have spoken to a GP about Long Covid problems that they have had. Those who had spoken to a GP about Long Covid (n = 213) were asked to indicate the extent which they agreed with the statement “*I am satisfied with the Long Covid care I have received from my GP.*” Responses to this were mixed albeit mostly skewed towards dissatisfaction: 24 (11.3%) patients strongly agreed, 36 (16.9%) agreed, 41 (19.2%) disagreed, 54 (25.4%) strongly disagreed, and 58 (27.2%) were neutral. Participants were asked to indicate their agreement with the statement, “*My GP was knowledgeable in relation to Long Covid*.” Many respondents (n = 127, 59.6%) strongly disagreed (n = 79, 37.1%) or disagreed (n = 48, 22.5%) with this statement. In contrast, 24 (11.3%) patients strongly agreed, 36 (16.9%) agreed, and 58 (27.2%) were neutral. Around half of respondents (n = 111, 52.4%) said their GP offered treatment to manage their Long Covid symptoms, with ‘*Medication*’ (n = 72, 49.3%) being the most common treatment offered. Participants were also asked “*Which of the following care initiatives would you like to see introduced by GPs for care of patients with Long Covid problems?*” and responses to this question are in Fig. 3.

**Fig. 4:**
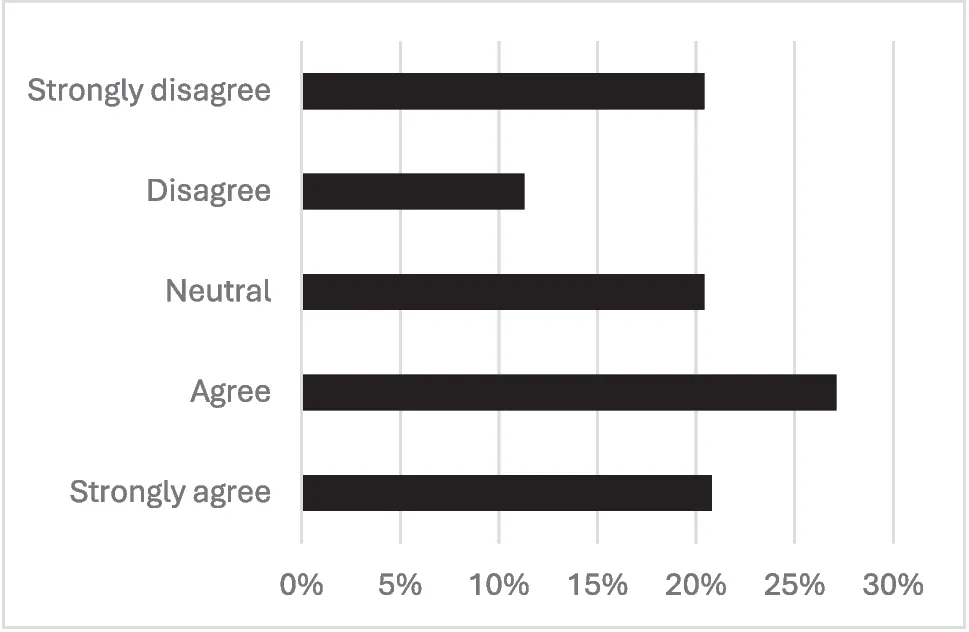
“I felt seen, heard, and understood by my general practitioner” (n = 221)

When asked to indicate the extent to which they agreed with the statement: “*With regards to Long Covid, having a good doctor-patient relationship between GPs and patients is very important.”*, 173 (77.9%) ‘Strongly agreed’, and 31 (14%) ‘Agreed’. Open responses elaborating on why they felt a good doctor-patient relationship was important (n = 179) highlighted the long-term, holistic, and empathetic qualities of positive GP/patient relationships, while noting potential negative relations arising from poor GP Long Covid knowledge, dismissiveness, and over-reliance on patients to self-manage their condition. Likewise, participants were asked to indicate the extent of their agreement with the statement: “*I felt seen, heard, and understood by my GP”,* but responses to this item were more mixed (Fig. 4). Optional open responses (n = 168) elaborating on this were largely negative with respondents highlighting feeling that their condition was misunderstood, poorly managed, overly psychologised, or not taken seriously by their GP, particularly with regards to issues like Long Covid aetiology, symptom management, and Long Covid knowledge. The more positive open responses to this statement highlighted the value of empathetic GPs that worked collaboratively with patients, listened to them, and validated their experiences.

**Fig. 5:**
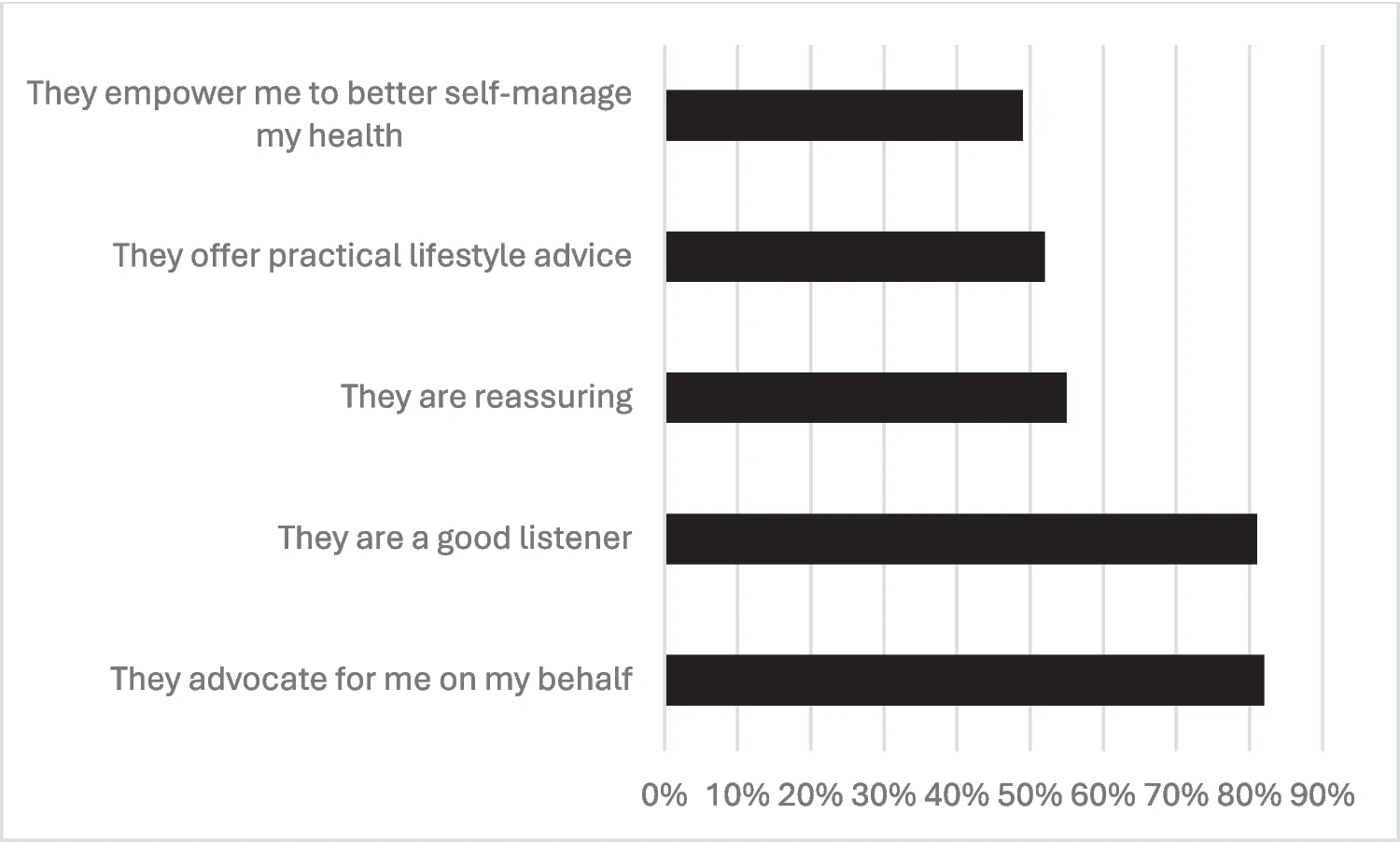
With regards to Long Covid care from general practitioners, please indicate which of these you think is important (n = 201)

Participants were asked to indicate with regards to Long Covid care from GPs, which supportive qualities they think are most important. GPs that were good listeners and advocated on patients’ behalf were particularly valued (Fig. 5). Optional open responses to this statement (n = 134) reinforced these findings but also stressed the importance of GP advice that is informed by a sufficient knowledge of Long Covid and its health impacts.

Beyond the primary care domain, there was also some data from open response questions that gave insight into participants’ perspectives of secondary care/hospital Long Covid services (n = 39) and Long Covid clinics (n-122). While many appreciated advanced treatments offered in hospitals, some reported long wait times and stressful experiences. Long Covid clinics were generally well-received for specialised care, but some respondents expressed frustration over limited availability and long wait times.

## Discussion

### Summary of key findings and their relationship to existing literature

This study aimed to cross-sectionally survey adults normally resident in the Republic of Ireland that have, or have had, Long Covid. Objectives included gaining a better understanding of this cohort’s demographic and medical characteristics, and their perspectives on Long Covid healthcare, especially general practice care.

Most survey respondents were female and middle-aged. These findings reiterate those of previous research saying that with regards to Long Covid, these groups are especially vulnerable [6–8]. However, this finding, particularly regarding the number of females (which was especially high), may be owing to other factors. For instance, some research has shown a greater tendency for women to take part in health surveys and seek health care. These confounding factors should be considered [27, 28]. The health profiles of the respondents were complex with many pre-existing comorbidities and Long Covid symptoms reported. This observed complexity reinforces previously made assertions that individuals living with Long Covid need ongoing holistic, multidisciplinary, and integrated care supports, the kind best provided by GPs working closely with a diverse team of health and social care professionals [2, 17]. Consistent with existing literature [29, 30], commonly reported comorbidities included those relating to sleep, mental health, and respiratory issues, alongside commonly reported Long Covid related conditions such as Postural Orthostatic Tachycardia Syndrome (POTS) and Chronic Fatigue Syndrome/Myalgic Encephalomyelitis (CFS/ME). Like the previous literature [29], respondents also reported that Long Covid has affected their physical health, psychological/emotional state, and quality of life, with fatigue/energy exertion and cognitive issues particularly evident. However, the symptom impact reported in this study was notably higher than usually found in general population studies [31–33]. Because our participants were largely recruited from an advocacy group, this cohort, like hospital-based samples, perhaps represents those most profoundly affected by Long Covid.

Almost all respondents consulted GPs for Long Covid care (n = 213, 95.9%), evidencing the crucial frontline role that GPs have been shown to play with regards to managing Long Covid, at least in the Republic of Ireland [2, 34]. Knowledgeable, attentive, and advocating GPs were especially valued. That around half of survey respondents were dissatisfied with GP Long Covid care and/or felt their GP lacked sufficient Long Covid knowledge was not surprising given that previous studies have also indicated this issue among healthcare workers [35–37]. The findings that attentive and supportive GPs were highly valued by patients are reminiscent of those in previous studies [17, 38], as are those which illustrate good acceptability of integrated, structured and patient-centred care supports [2, 17].

### Strengths and limitations

Recruiting research participants living with Long Covid from general practices in Ireland has been challenging in the past [17]. This has mostly been the case because Long Covid diagnoses are often difficult to find on practice database systems due to them not routinely being reported and/or clearly recorded (e.g., via designated coding systems like ICPC-2). However, there was a positive response to this study’s survey (n = 222), so much so that the response numbers were deemed satisfactory by the research team after only 20 days of the survey being online, and this is largely due to the fact that the choice was made to recruit participants from a well-established and populated Long Covid advocacy group. The online aspect of the survey was another good methodological choice. While the survey was not accessible to people not using the internet, a notable flaw, its response numbers show that it was accessible to many people, more people than it likely would have been possible to recruit through general practice sites. However, the survey did take some participants a long time to complete, and this in combination with Long Covid related fatigue among respondents, was mentioned as a barrier to taking part. Also, another potential limitation was the representativeness of the sample surveyed. As participants were largely recruited from an advocacy group, one might argue that the sample was not reflective of typical patients attending general practice with Long Covid. Additionally, the findings reflect only the perspectives of patients in the Republic of Ireland. While this is appropriate given the study’s focus, it limits how well the results represent patients internationally. Lastly, like all cross-sectional designs, this study showed good feasibility and descriptive power but was limited in that it was unable to measure the topic longitudinally or demonstrate causality.

### Implications for research and/or practice

The important role and considerable potential of GPs in Long Covid care have been well established. However, there is room for improvement, and future policy, practice, and research should prioritise implementing and evaluating interventions to maximise the potential of GP Long Covid care. Greater activity around Long Covid education and training initiatives [35, 39] for GPs would be particularly useful. Such training might benefit most from including material highlighting the care needs and concerns of people living with Long Covid, as well as GP guidance on investigative, treatment, and referral supports. Also, and especially in the context of overburdened health services, utilising existing structured programmes developed for other chronic illnesses may be helpful. However, programme adaptation and Long Covid specific training for programme staff will likely be required. For example, a potentially suitable initiative in the Republic of Ireland called the ‘Living Well Programme’ [40] would need to be adapted as it contains exercise components that could pose risks for people experiencing Post Exertional Malaise (PEM), a common Long Covid symptom affecting 92% of the people in this survey.

## Conclusion

There are still many people burdened with Long Covid in the Republic of Ireland. Their care needs are often complex, profound, and disabling. Delivery and evaluation of continual, holistic and integrated multidisciplinary supports, such as those provided by GPs and integrated primary/secondary care supports, is needed. Ongoing efforts to provide education and training for GPs, as well as community-focused structured care programmes, may help ensure that people living with Long Covid are not missed and that their health needs are attended to.

## Supplementary Information

Below is the link to the electronic supplementary material.

Supplementary file1 (DOCX 32 KB)

## Data Availability

Aggregated anonymous data outlining the study’s findings is available at at https://doi.org/10.5281/zenodo.20607521. Data is presented in this format to ensure participants’ confidentiality. Access to pseudonymised individual level data is available on request from the corresponding author.
