# Supplementary material for: Long covid and general practice: a survey of patients in the Republic of Ireland
Source: Ir J Med Sci. 2026 Jun 13;195(5):2525–33. doi: 10.1007/s11845-026-04481-1 (PMC13739935; doi:10.1007/s11845-026-04481-1)
Supplement: Supplementary file 1 [file 11845_2026_4481_MOESM1_ESM.docx]

**Long Covid and General Practice: A Survey of Patients in the Republic of Ireland**

***Irish Journal of Medical Science***

John Broughan^1^, Yao Xie^2^, Crea Carberry^2^, Sarah O’Connell^3^, Jacinta Fay^3^, Pamela Morrison^3^, Nandakumar Ravichandran^2^, Stefano Savinelli^2,4^, Geoff McCombe^2^, Mary Higgins^2, 5^, John S Lambert^2,6,7^, Walter Cullen^2^

**Corresponding author:** John Broughan ([john.broughan@ucd.ie](mailto:john.broughan@ucd.ie))

**Supplementary material**

***Study instrument***

1. What is your age?

- Under 18
- 18-24
- 25-34
- 35-44
- 45-54
- 55-64
- 65-74
- 75-84
- 85+

2. Do you normally live in the Republic of Ireland?

- Yes
- No

3. What is your gender?

- Male
- Female
- Non-binary
- Prefer to self-describe: _____________________________
- Prefer not to say

4. Country of Birth?

- Republic of Ireland
- Other EU
- Non-EU

5. Does not being born in the Republic of Ireland impact your ability to access healthcare for your condition?

- Yes
- No

6. How many times did you have COVID-19?

- 0
- 1
- 2
- 3
- 4
- 5+

7. Did you ever have a COVID-19 diagnosis confirmed by a PCR/Antigen tests or qualified health professional?

- Yes
- No

8. Do you have Long Covid, or have you had Long Covid in the past?

- Yes
- No

9. Who raised the possibility that you might have Long Covid?

- I did
- A family member/friend
- A health professional
- Other (please explain)

10. If it was you who raised the possibility that you might have Long Covid, who or what led you to believe it might be Long Covid?

- Friends
- Family
- Patient community
- Internet research
- Long Covid charity group
- Other (please explain)

11. Did you have a Long Covid diagnosis confirmed by a qualified health professional?

- Yes
- No

12. If you had COVID-19 multiple times, which COVID-19 infection do you think triggered Long Covid?

- 1^st^
- 2^nd^
- 3^rd^
- 4^th^
- 5^th^
- Other (please explain)

13. When did you have the COVID-19 infection that you think triggered Long Covid? Please be as specific as you can be.

Day/Month/Year

14. Are you diagnosed with, or have you been diagnosed with in the past, any of these health conditions? (Tick all that apply)

- A different post-viral fatigue syndrome
- Chronic Fatigue Syndrome / Myalgic encephalomyelitis
- Glandular fever
- Lupus and Rheumatoid Arthritis
- Multiple Sclerosis (MS)
- POTS (Postural Orthostatic Tachycardia Syndrome
- Asthma or COPD
- Interstitial lung disease
- Anxiety
- Depression
- Post-Traumatic Stress Disorder (PTSD)
- Myocarditis or Pericarditis
- Heart failure or deconditioning
- Thyroid disorders (Hypothyroidism/Hyperthyroidism)
- Diabetes
- Sleep apnoea or insomnia

15. Did your GP suggest that your physical symptoms might be caused by psychological issues such as stress and anxiety?

- Yes
- No

16. Please indicate how much you agree with the following statement:
    “The first 2-3 weeks of having COVID-19 made me feel unwell.”

- Strongly disagree
- Disagree
- Neutral
- Agree
- Strongly agree

17. Please indicate how much you agree with the following statement:
    “Long Covid made me feel unwell”

- Strongly disagree
- Disagree
- Neutral
- Agree
- Strongly agree

18. Please indicate the approximate length of time that you have experienced Long Covid issues.

- 4-12 weeks
- 3 to 6 months
- 6 months to 1 year
- More than 1 year
- More than 2 years
- More than 3 years
- More than 4 years

19. If you don’t have Long Covid now but you had it in the past, how long ago did you have Long Covid?

- Less than six months ago
- Six to twelve months ago
- More than one year ago
- More than two years ago
- More than three years ago
- More than four years ago

20. Which of these symptoms have you experienced because of having Long Covid?
    (Tick all that apply)

- Fatigue / tiredness
- Post exertional malaise (Extreme fatigue after activity)
- Shortness of breath
- Cognitive issues (For example, problems with memory, concentration, slow thinking, brain fog)
- Dysautonomia (Autonomic nervous system dysfunction) / POTS or Postural Orthostatic Tachycardia Syndrome (Rapid heart rate upon standing)
- Heart palpitations
- Dizziness
- Joint pain
- Mobility issues
- Exertion intolerance
- Changes to smell / taste
- Chest pain / tightness
- Insomnia / Difficulty sleeping
- Pins and needles
- Depression
- Anxiety
- Tinnitus / earaches
- Cough
- Gastrointestinal problems (stomach, intestines)
- Headaches
- Migraines
- Sore throat
- All the above
- None of the above
- Other (please explain)

21. Please indicate how much you agree with the following statement:
    “Long Covid negatively impacted my physical health”

- Strongly disagree
- Disagree
- Neutral
- Agree
- Strongly agree

22. If relevant, please briefly explain how Long Covid negatively impacted your physical health.

__________________________________________________________________________________________________________________________________________

23. Please indicate how much you agree with the following statement:
    “Long Covid negatively impacted my quality of life.”

- Strongly disagree
- Disagree
- Neutral
- Agree
- Strongly agree

24. If relevant, please briefly explain how Long Covid negatively impacted your quality of life.

__________________________________________________________________________________________________________________________________________

25. Please indicate how much you agree with the following statement:
    “Long Covid negatively impacted my mental health.”

- Strongly disagree
- Disagree
- Neutral
- Agree
- Strongly agree

26. If relevant, please briefly explain how Long Covid negatively impacted your mental health.

__________________________________________________________________________________________________________________________________________

27. Have you spoken to a General Practitioner (GP) about Long Covid problems that you have had?

- Yes
- No

28. If you have spoken to a GP about Long Covid problems, please indicate how much you agree with the following statement:
    “I am satisfied with the Long Covid care I have received from my GP.”

- Not applicable
- Strongly disagree
- Disagree
- Neutral
- Agree
- Strongly agree

29. If you have spoken to a GP about Long Covid problems, please indicate how much you agree with the following statement:
    “My GP was knowledgeable in relation to Long Covid.”

- Not applicable
- Strongly disagree
- Disagree
- Neutral
- Agree
- Strongly agree

30. Did your GP offer treatment to manage Long Covid symptoms?

- Yes
- No

31. If yes, what treatment(s) did they offer? (Tick all that apply)

- Medication
- Supplements
- Exercise
- Other (please explain)

32. Which of the following would you like to see introduced by GPs for care of patients with Long Covid problems?
    (Tick all that apply)

- More patient input / involvement in the care process
- Use of Long Covid tools (For example, questionnaires and scales) to assess and monitor Long Covid symptoms over time.
- Better links with hospital specialists (For example, Cardiologists, Respiratory Physicians etc.)
- Better links with Long Covid clinics.
- Better links with other services that might help (For example, Psychologist/Counselling, Occupational Therapy, Home Help, Physiotherapy)
- All the above
- None of the above

33. Please state how much you agree with the following statement:
    "With regards to Long Covid, having a good doctor-patient relationship between GPs and patients is very important."

- Strongly disagree
- Disagree
- Neutral
- Agree
- Strongly agree

34. Please explain your answer to the last question. Why do you think this?

_________________________________________________________________________________________________________________________________________

35. Please state how much you agree with the following statement:
    "I felt seen, heard, and understood by my GP”

- Strongly disagree
- Disagree
- Neutral
- Agree
- Strongly agree

36. Please explain your answer to the last question. Why did you choose these options?

__________________________________________________________________________________________________________________________________________

37. With regards to Long Covid care from GPs, please indicate which of these you think is important (Tick all that apply).

- They are reassuring
- They offer practical lifestyle advice
- They empower me to better self-manage my own health
- They are a good listener
- They advocate for me on my behalf

38. Please explain your answer to the last question. Why did you choose these options?

__________________________________________________________________________________________________________________________________________

39. Are there any other care strategies you would recommend for GPs in managing Long Covid? Please explain why you recommend these. __________________________________________________________________________________________________________________________________________
